# Death from Nitrous Oxide

**Published:** 1880-05

**Authors:** 


### DEATH FROM NITROUS OXIDE.

A DEATH from inhalation of nitrous oxide gas occurred lately at Exeter, England. The gas was given to produce insensibility during the extraction of a tooth. The patient was a woman of about forty years of age. After a few inhalations the pulse was noticed to become weak, and the administration was stopped for a time. As the patient had not become insensible, the inhalation was resumed, and the tooth withdrawn. The patient became livid, and in a few minutes died. Her health had been excellent previously, and there was no reason known why she would not be a good subject for the gas. A case has also been reported in this country.—*Boston Med. and Surg. Journal.*

